# Missing ligand model in autologous stem cell transplantation

**DOI:** 10.1038/sj.bjc.6604153

**Published:** 2007-12-18

**Authors:** M Stern, M Paulussen, J Rischewski, A Tichelli, A Gratwohl

**Affiliations:** 1 Department of Hematology, Basel University Hospital, Petersgraben 4 Basel, Basel CH-4301, Switzerland; 2 Department of Hematology/Oncology, University Children's Hospital, Basel CH-4005, Switzerland

**Sir**,

We read with interest the article of Leung *et al* (2007) describing a reduced relapse rate after autologous HSCT in patients with an inhibitory KIR–HLA mismatch treated with autologous transplantation for non-Hodgkin's lymphoma or solid tumour. The analysis was performed after several recent publications indicated a reduced relapse rate in patients treated by allogeneic HSCT (by the same group (Leung *et al*, 2005) and others (Hsu *et al*, 2005)) in the presence of a mismatch between donor inhibitory KIR and patient HLA.

The manuscript prompted us to analyse the outcome after autologous transplantations carried out between 1997 and 2006 at our centre. Patients were included if HLA typing allowed unequivocal deduction of the KIR-ligand status, and were grouped according to the presence or absence of KIR ligands, without taking into account inhibitory KIR. This type of approach is generally chosen for retrospective studies (Miller *et al*, 2007), as population frequencies of KIR gene distribution indicate that virtually all Caucasians possess and express *KIR2DL1* and *KIR2DL2/3* and only a minority do not possess or express *KIR3DL1*. Therefore, grouping according to KIR ligands should correctly classify the vast majority of patients regarding an inhibitory KIR/HLA mismatch (in the publication by Leung, 1 out of 16 patients would have been misclassified using this approach).

Outcome of 67 autologous transplants for solid tumour (*N*=23) or lymphoma (*N*=44) was analysed. In 21 patients, grafts were depleted from tumour cells and T cells by CD34 selection. Absence of KIR ligands had no effect on disease-free survival (DFS) (relative risk (RR) 1.03 *vs* patients with all KIR ligands, *P*=0.91) or disease relapse (RR 1.24, *P*=0.23; Figure 1). Similar results were seen when the analysis was restricted to patients receiving T-cell-depleted transplants (RR for DFS 1.08, *P*=0.88; RR for relapse 1.15, *P*=0.79). Excluding patients missing Bw4 (the ligand for *KIR3DL1,* the only inhibitory KIR absent in a significant proportion of Caucasians), the relative risks for DFS and relapse were 1.15 (*P*=0.59) and 1.36 (*P*=0.28) for missing ligand patients.

In conclusion, we were not able to show a missing KIR-ligand effect in our population of autologous HSCT recipients. One caveat of our approach is that a missing KIR ligand does not imply an inhibitory KIR/HLA mismatch, however, restriction of analysis to the almost universally expressed KIR2D did not show significant differences. We therefore believe that while the data presented by Leung *et al* (2007) are intriguing, they should be confirmed in large studies.

## Figures and Tables

**Figure 1:**
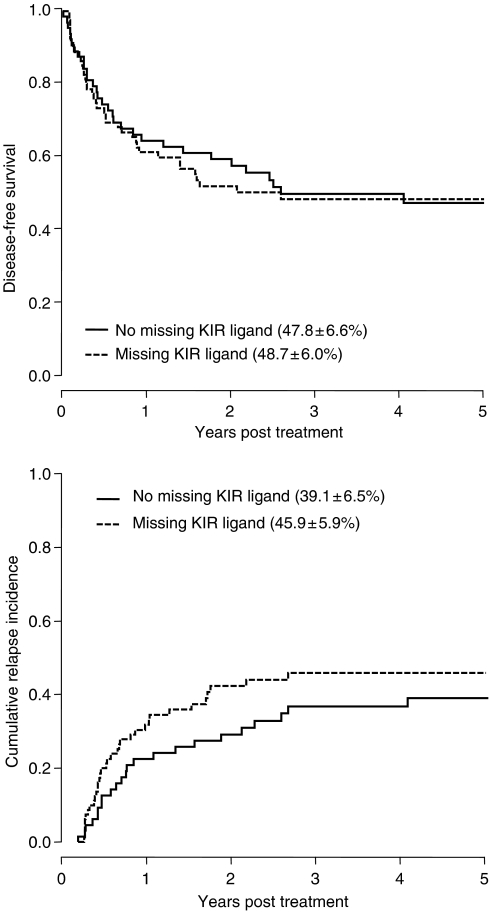
Disease-free survival (upper panel) and cumulative incidence of relapse (lower panel) are not different between patients with or without a missing KIR ligand after high-dose chemotherapy and autologous haematopoietic stem cell transplantation.
